# P033: Incidence estimate of Clostridium difficile infection in Emilia-Romagna Region by linkage of administrative and laboratory data

**DOI:** 10.1186/2047-2994-2-S1-P33

**Published:** 2013-06-20

**Authors:** M Morandi, R Buttazzi, M Marchi, F Morsillo, C Gagliotti, ML Moro

**Affiliations:** 1 Social Health Agency of Emilia-Romagna Region, Bologna, Italy

## Objectives

To estimate the incidence of *Clostridium difficile* infection (CDI) in Emilia-Romagna Region (ER) by linking data of Patient Hospital Discharge Form (PHDF) and microbiological labs.

## Methods

We linked regional data of PHDF and labs by anonymous patient code and we considered only public hospitals with at least 1 identified positive lab test for CD. CDI was defined as follows: “*Confirmed case*” (*Cc*) where a specific ICD9 code (00845) was present on the PHDF or an unspecific code for diarrhoea (or intestinal infection) and positivity for CD toxin was retrieved from the lab; “*Probable case*” (*Pc*) where either a specific ICD9 code without positivity for CD toxin test or exclusively CD toxin positivity was present. We considered only people older than 1 year and incidence was calculated both for all inpatients and for residents in ER. By linking dates of hospital admission and discharge and date of lab testing we defined CDI as hospital-acquired (HA: >2 days from admission within 28 day from discharge), community-acquired (CA: within 2 days from admission and after 84 days from possible previous discharge, or none admission) or indeterminate (IA: all the remaining cases with hospitalization and lab test).

## Results

For 2011, 980 CDI (41.5% were *Cc*) were identified in 27 ER public hospitals accounting for more than 2.6 millions hospital-days (65% of ER hospital-days). Most cases were resident in ER (92.2%), female (57.2%) and old (median age = 80). Of the total cases, 43.5% had only CD toxin positivity without a specific or unspecific ICD9 code for CDI and 15.0% had only a specific code without lab confirmatory data. The overall incidence among the population older than 1 year was 13.7 per 100,000 inhabitants for *Cc* and 31.8 for *Cc* and *Pc*. The HA incidence density was 2.7/10,000 patient-days. The place of transmission for residents in ER was HA in 76.1% of cases and CA in 9.4%; 5.2% were IA and 9.3% were not attributable because of lacking of lab results.

## Conclusion

The linkage of administrative data allows to improve incidence estimates of CDI in ER, yielding values in line with European data. It also allows to assess the locus of CD transmission, which is predominantly the hospital, though community CDI accounted for almost one tenth of cases.

## Disclosure of interest

None declared

